# Supplementation of xylo-oligosaccharides to suckling piglets promotes the growth of fiber-degrading gut bacterial populations during the lactation and nursery periods

**DOI:** 10.1038/s41598-022-15963-4

**Published:** 2022-07-08

**Authors:** Francesc González-Solé, David Solà-Oriol, Yuliaxis Ramayo-Caldas, Maria Rodriguez-Prado, Gemma González Ortiz, Michael R. Bedford, José Francisco Pérez

**Affiliations:** 1 Animal Nutrition and Welfare Service (SNIBA), Department of Animal and Food Science, Autonomous University of Barcelona, 08193 Bellaterra, Spain; 2 Animal Breeding and Genetics Program, Institute for Research and Technology in Food and Agriculture (IRTA), Torre Marimon, 08140 Caldes de Montbui, Spain; 3 AB Vista, Marlborough, SN8 4AN Wiltshire UK

**Keywords:** Molecular biology, Microbiology, Microbial genetics, Dysbiosis, Diarrhoea, Animal physiology

## Abstract

Modulating early-life microbial colonization through xylo-oligosacharides (XOS) supplementation represents an opportunity to accelerate the establishment of fiber-degrading microbial populations and improve intestinal health. Ninety piglets from 15 litters were orally administered once a day from d7 to d27 of lactation with either 5 mL of water (CON) or 5 mL of a solution containing 30 to 60 mg of XOS (XOS). Supplementation ceased at weaning (d28) when all piglets were fed the same commercial pre-starter diet. Growth performance did not differ between treatments during the experimental period (d7 to d40). Piglet’s fecal microbiota (n = 30) shifted significantly from the end of lactation (d27) to nursery period (d40) exhibiting an increase in microbial alpha diversity. Animals supplemented with XOS showed higher richness and abundance of fiber-degrading bacteria and short-chain fatty acid (SCFA) production at d27 and d40. Additionally, the predicted abundance of the pyruvate to butanoate fermentation pathway was increased in the XOS group at d40. These results show that supplementation of XOS to lactating piglets promotes fiber-degrading bacterial populations in their hindgut. Moreover, differences observed in the nursery period suggest that XOS can influence the microbiota in the long-term.

## Introduction

Weaning is considered a critical period for piglets, when they suddenly need to deal with different stressful events. The challenge includes an abrupt diet change from sow’s milk to plant-based dry solid diets, which contains many ingredients and vegetable structures that piglets have not eaten before^1^. Weaning also occurs when their gut barrier is still developing, which can induce long-lasting deleterious consequences on gut health^2^. As a result, growth performance of the pigs is impaired and there is a high risk of appearance of post-weaning diarrhea, which is associated with enormous economic losses for the pork industry^3^.

The gut microbiota during early-life is considered to have a decisive role in the development and programming of both the mucosal immune response and the establishment of the adult microbiota^4–7^. There is increasing evidence that the existence of a “window of opportunity” occurs in the early life of animals when an intervention may determine an improvement of microbial colonization and, hence, benefit the immunity status of the pig^8,9^.

Multiple management and feeding strategies in the early stages of weaning have been explored in order to take advantage of the plasticity of the microbiota during that period, the goal being to mitigate the negative impact of weaning and improve post-weaning performance^10,11^. One such strategy involves providing dietary fiber to suckling piglets to accelerate gut microbiome maturation, thus increasing populations capable of breaking down complex polysaccharides before they have to deal with the post weaning feed^12–14^. Other studies examined the oral supplementation of prebiotics, such as fructo-oligosacharides or galacto-oligosacharides, to suckling piglets to enable identifying a modulation of the gut microbiota, intestinal morphology and function^11^.

Among dietary fiber compounds, xylo-oligosaccharides (XOS) are oligomers composed of 2–6 xylose units linked through β-(1 → 4)-linkages^15^. Their selective fermentation has been shown to induce changes in both composition and activity of the gastrointestinal microbiota, stimulating the growth of butyrate-producing bacteria^16,17^. Butyrate has been associated with benefits in intestinal health, such as the modulation of the mucosal inflammatory response and barrier function^18,19^. In fact, several studies supplementing XOS into weaning pig^20–22^ and broiler^23–25^ diets have demonstrated such improvements in gut health, and in some cases these benefits have been associated with enhanced growth performance^20,23^. Overall, the beneficial effects of XOS fulfill the definition of prebiotic, although their mechanism of action seems to be specific. Their effects have been observed at supplementation levels of 0.1–0.2 g/kg, which are considered too low to be effective through its quantitative fermentation alone^21,22,25^. That is why it has been proposed to describe XOS as a “stimbiotic”, which is a new term defined as a “non-digestible but fermentable additive that can stimulate a fiber-degrading microbiome to increase fiber fermentability at doses which clearly are too low to contribute in a meaningful manner to short-chain fatty acid (SCFA) production”^26^. The increase in fiber fermentability may contribute to more oligosaccharide production, and therefore, SCFA production, which is considered an indirect mechanism of action to explain part of its health-related benefits^27^. Nevertheless, little research has been conducted on the effects of XOS supplementation in the early stages of a piglet’s life.

This trial hypothesized that providing low doses of XOS to suckling piglets modulates the early-colonization of the piglet intestinal tract by stimulating the growth of fiber-degrading and butyrate-producing bacteria, and conditioning the establishment of a differential microbiota population in the hindgut after weaning. Therefore, the objective was to explore if daily supplementation of XOS to suckling piglets influences the fecal microbiota composition and its functionality at the end of the suckling period, but also during the nursery period when piglets were exposed to the same diet and same environmental conditions.

## Results

### Growth performance

The effects of XOS supplementation on growth performance of piglets are summarized in Table 1 and individual data of growth performance can be found as Supplementary Table S1. No differences in body weight (BW) or average daily gain (ADG) were observed in any of the studied periods or the overall (*P* > 0.05).

**Table 1:** Effects of the XOS supplementation on growth performance.

Items^a^	Experimental groups^b^		SEM	*P* value^c^
	CON	XOS		
BW d7, kg	2.91	2.91	0.064	0.934
BW d28, kg	7.55	7.48	0.182	0.772
BW d40, kg	10.02	9.83	0.264	0.598
ADG d7–d28, kg	0.221	0.218	0.0077	0.782
ADG d28–d40, kg	0.195	0.196	0.0173	0.968
ADG d7–d40, kg	0.216	0.210	0.0074	0.604

^a^*BW* Body weight, *ADG* Average daily gain.

^b^*CON* Piglets supplemented with water, *XOS* Piglets supplemented with XOS.

^c^*P* value obtained from ANOVA test.

### Microbiota structure and biodiversity

An average of 19,210 ± 9042 high-quality reads were generated per sample after quality control, from 60 fecal samples. The obtained OTU raw counts are available as Supplementary Table S2. Microbiota community alpha diversity measured as observed OTUs and Shannon index are summarized in Table 2. No interactions were observed between treatments and sampling days. Higher richness values were obtained in both observed OTUs and Shannon index on the last sampling day (*P* < 0.001). In addition, piglets supplemented with XOS showed more OTUs when the treatment was analyzed as a main factor (*P* = 0.019).

**Table 2:** Effect of XOS supplementation on observed OTUs and Shannon indexes from the analysis of the fecal microbiota.

Item	Observed OTUs	Shannon
**Treatment effect**^a^		
CON	128.8	3.92
XOS	155.1	4.06
SEM	9.27	0.081
**Day effect**		
d27	115.6	3.73
d40	168.3	4.24
SEM	9.27	0.081
***P* value**^b^		
Treatment	**0.019**	0.150
Day	**< 0.001**	**< 0.001**
Treatment × day	0.5691	0.459

Significant values are in bold.

^a^*CON* Piglets supplemented with water, *XOS* Piglets supplemented with XOS.

^b^*P* value obtained from ANOVA test.

**Figure 1:**
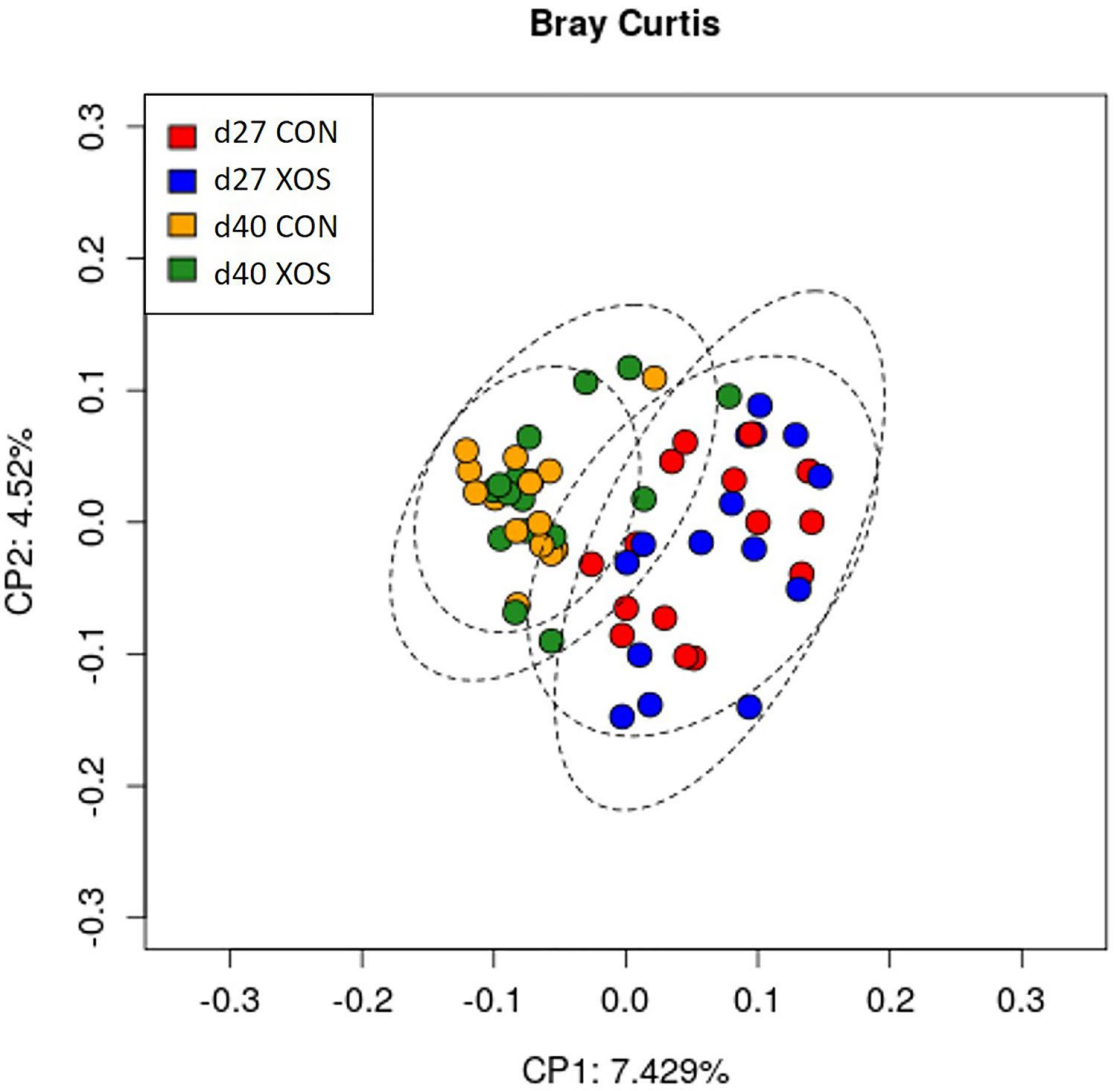
Principal coordinate analysis (PCoA) ordination performed by Bray–Curtis dissimilarities representing the microbiome composition for all animals. d27 CON (red): fecal samples of piglets supplemented with water on day 27; d27 XOS (blue): fecal samples of piglets supplemented with XOS on day 27; d40 CON (yellow): fecal samples of piglets supplemented with water on d40; and d40 XOS (green): fecal samples of piglets supplemented with XOS on d40.

Beta diversity analysis at the OTU level using the Bray–Curtis distance revealed that the microbiota structure changed from d27 to d40 (*P* < 0.001). No differences were attributed to treatment as the main effect. However, pairwise PERMANOVA revealed that XOS and CON tended to have a different diversity composition on d40 (*P* = 0.076). The Principal Component Analysis (PCoA) based on the Bray–Curtis distance matrix shows a distinctive clustering corresponding to the two sampling days (Fig. 1).

### Composition of gut microbiota

**Figure 2:**
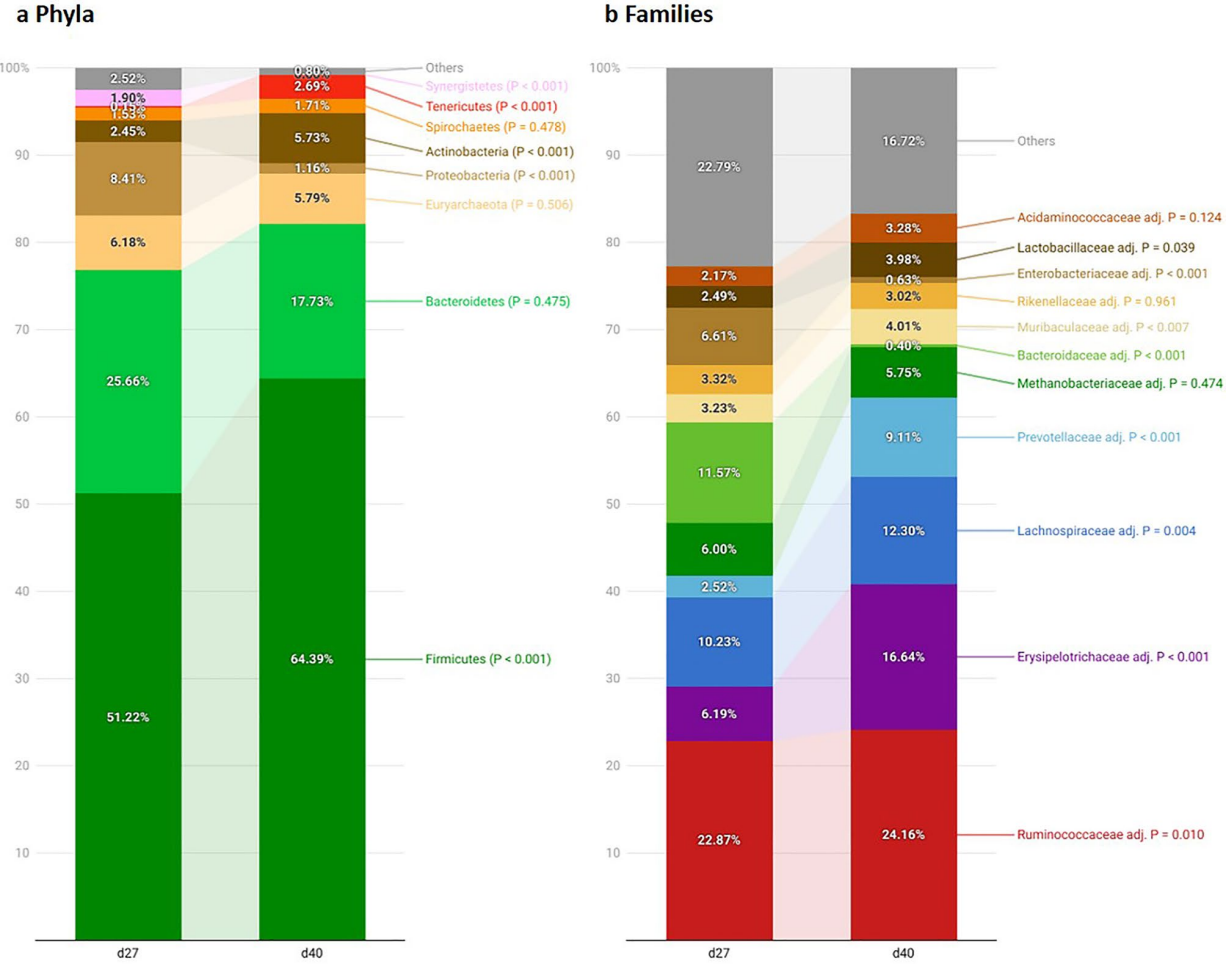
Relative abundances (RA) of the main phyla (**a**) and families (**b**) observed in the analysis of the microbiota of piglets by massive sequencing of the 16S rRNA gene. Figure created with the online open-source tool Datawrapper (http://datawrapper.de).

The relative abundance of the main phyla and families observed are presented in Fig. 2. The predominant phyla were Firmicutes (58%) and Bacteroidetes (22%) followed by Euryarchaeota (6%), Proteobacteria (5%) and Actinobacteria (5%), representing together 96% of the fecal microbiome. At the family level, *Rumminococcaceae* (24%), *Erysipelotrichaceae* (11%) and *Lachnospiraceae* (11%) were the predominant groups.

From d27 to d40, the relative abundance of Firmicutes increased from 51 to 64% (*P* < 0.001) while Bacteroidetes numerically decreased (26% to 18%; *P* = 0.475). The relative abundance of Proteobacteria decreased (8% to 1%; *P* < 0.001) and Actinobacteria increased (2% to 6%; *P* < 0.001) with age. At the family level, an increase of four predominant groups was identified, including *Ruminococcaceae* (*P* = 0.01), *Erysipelotrychaceae* (*P* < 0.001), *Lachnospiraceae* (*P* = 0.004) and *Prevotellaceae* (*P* < 0.001). Meanwhile, the relative abundance of the *Bacteroidaceae* family significantly dropped (*P* < 0.001).

**Figure 3:**
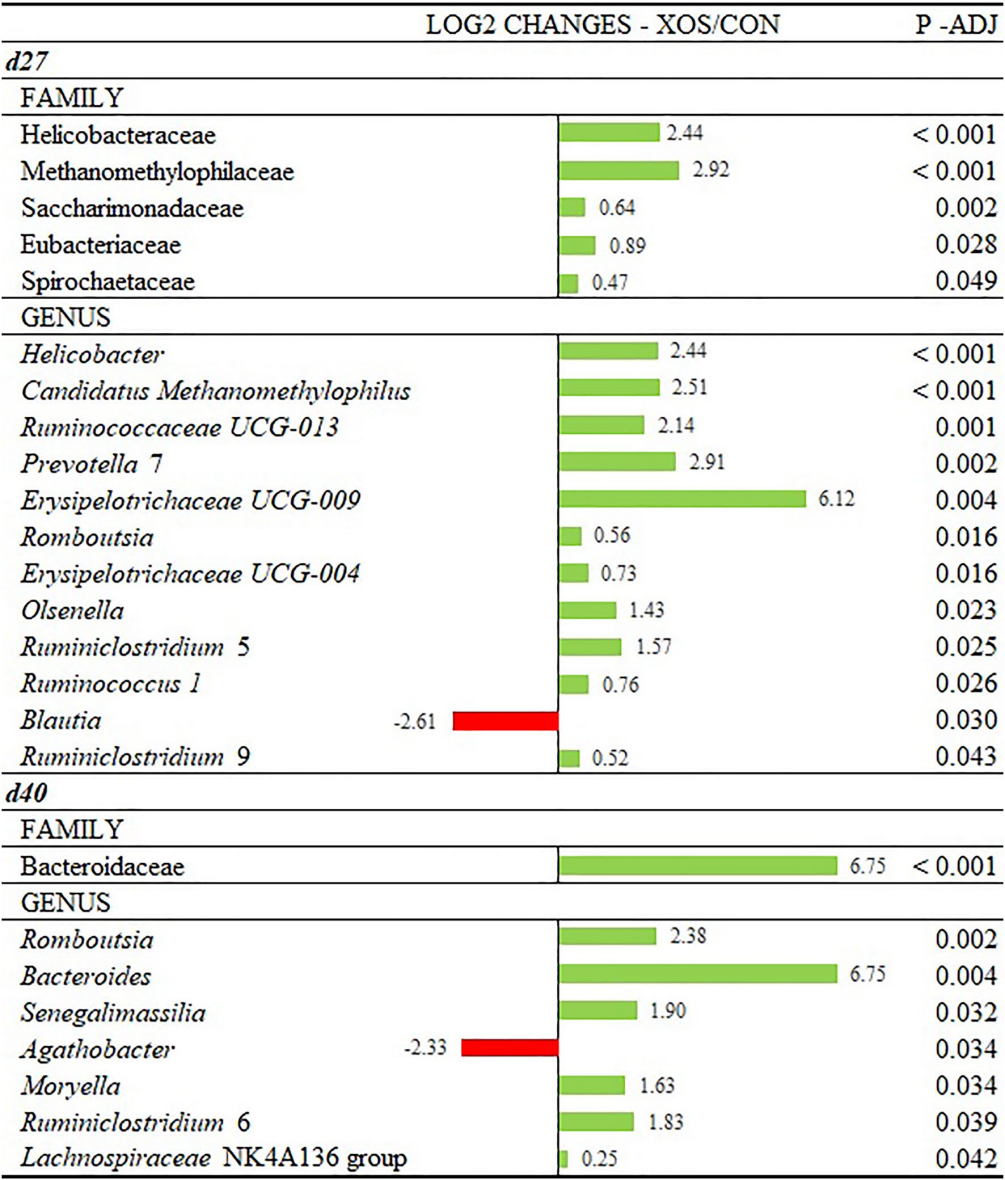
Log_2_ changes promoted by xylo-oligosacharides supplementation (fold discovery rate p-adjusted < 0.05) in microbial families and genera on d27 and d40. Taxa are sorted by level of significance (from higher to lower). Differences presented are based on all taxa detected in samples per treatment.

Log_2_ fold changes were calculated for the taxa that showed significant differences among groups at the family and genus levels (Fig. 3). On d27, XOS supplementation promoted the proliferation of families *Helicobacteraceae*, *Methanomethylophilaceae*, *Saccharimonadaceae*, *Eubacteriaceae* and *Spirochaetaceae* compared to CON piglets. At the genus level, *Helicobacter*, *Candidatus Methanomethylophilus*, *Ruminococcaceae* UCG-013, *Prevotella* 7, *Erysipelotrichaceae* UCG-009, *Romboutsia*, *Erysipelotrichaceae* UCG-004, *Olsenella*, *Ruminiclostridium* 5, *Ruminococcus* 1 and *Ruminiclostridium* 9 were increased compared to CON piglets (*P* < 0.05). In contrast, *Blautia* genus was significantly lower in the XOS group compared to CON (*P* = 0.030). On d40, XOS supplementation increased the family taxon *Bacteroidaceae* and also the genus *Romboutsia*, *Bacteroides*, *Senegalimassilia*, *Agathobacter*, *Moryella*, *Ruminiclostridium* 6 and *Lachnospiraceae* NK4A136 group (*P* < 0.05). The relative abundance of *Agathobacter* genus was lower in XOS-supplemented piglets (*P* = 0.034).

### Predicted functionality of the gut microbiota

**Figure 4:**
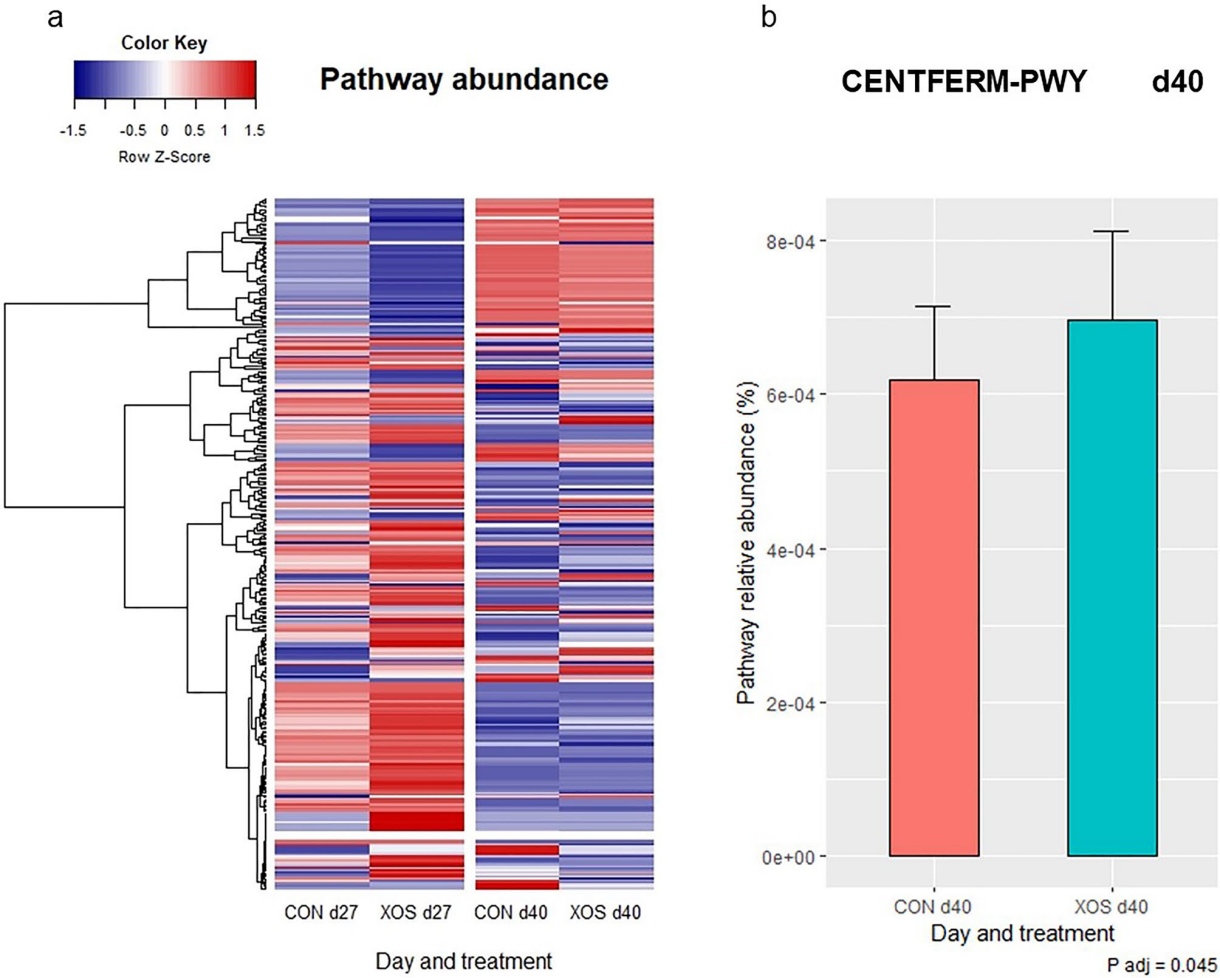
Predicted functionality of the fecal microbiota. The potential functionality of the gut microbiota was inferred from 16S rRNA gene amplicon sequences in feces collected from control piglets (CON) and piglets supplemented with xylooligosacharides (XOS) at the end of lactation period (d27) and during the nursery period (d40). (**a**) Heatmap representing the mean relative abundance of each predicted pathways in each treatment. The color represent the Z-scores (row-scaled relative abundance) from low (blue) to high values (red). Predicted pathways (rows) were clustered by the average method. (**b**) Bar plot representing the relative abundance of the CENTFERM-PWY predicted pathway at d40. Error bar represents standard error of the mean. d27 CON: fecal samples of piglets supplemented with water on day 27; d27 XOS: fecal samples of piglets supplemented with XOS on day 27; d40 CON: fecal samples of piglets supplemented with water on d40; and d40 XOS: fecal samples of piglets supplemented with XOS on d40. Figure created by using open-source software R v4.2.0. (https://www.r-project.org/foundation/) and the gplots package (https://cran.r-project.org/web/packages/gplots/).

The functional capacity of the gut microbiota was predicted by using PICRUSt2 based on 16S rRNA gene amplicon sequences. Raw counts of predicted pathways can be found as Supplementary Table S3. A heatmap representation of all predicted pathways revealed major differences of functional potential of the microbiota between d27 and d40, while the differences between CON and XOS animals were subtle (Fig. 4). A more in-depth analysis of the abundance differences in the pathway metabolic data between both treatment groups showed that 186 out of 349 pathways significantly changed from d27 to d40, while supplementation of XOS significantly changed the relative abundance of 3 and 24 pathways out of 349, at 27 and 40 days of age, respectively. Interestingly, the results showed an increase of pathway CENTFERM-PWY, also known as “pyruvate fermentation to butanoate”, in XOS-supplemented animals on d40 (*P* = 0.045).

### Short-chain fatty acids analysis

The SCFA in the fecal samples on d27 and d40 are summarized in Table 3 and the individual profile can be found in Supplementary Table S4. No interactions were observed for any of the SCFA measured (*P* > 0.05). Supplementation with XOS did not influence SCFA (*P* > 0.05). Feces of piglets of 40 days of age had higher concentrations of total SCFAs, acetic acid, propionic acid, butyric acid, lactic acid and volatile fatty acid (*P* < 0.001).

**Table 3:** Effect of XOS supplementation in total SCFAs concentration in feces, expressed in mM.

Item^a^	Total SCFAs	Acetic A	Propionic A	Butyric A	Valeric A	Lactic A	BCFAs	VFAs
**Treatment effect**^b^								
CON	81.62	45.05	14.94	11.00	2.43	1.43	6.81	80.17
XOS	84.71	48.43	15.81	10.11	2.64	1.06	6.67	83.66
SEM	4.912	2.070	1.243	1.315	0.362	0.234	0.683	4.955
**Day effect**								
d27	60.91	34.62	8.67	7.18	2.31	0.51	7.65	60.38
d40	105.42	58.86	22.08	13.93	2.76	1.98	5.83	103.45
SEM	4.683	2.094	1.132	1.277	0.318	0.238	0.737	4.709
***P* value**^c^								
Treatment	0.657	0.253	0.621	0.634	0.684	0.266	0.889	0.620
Day	** < 0.001**	** < 0.001**	** < 0.001**	** < 0.001**	0.242	** < 0.001**	0.105	** < 0.001**
Treatment x day	0.605	0.917	0.703	0.266	0.944	0.808	0.280	0.597

Significant values are in bold.

^a^*SCFAs* Short-chain fatty acids, *BCFAs* Branched-chain fatty acids, *VFAs *Volatile fatty acids.

^b^*CON* Piglets supplemented with water, *XOS* Piglets supplemented with XOS.

^c^*P* value obtained from ANOVA test.

## Discussion

Supplementing monogastric animals with XOS has proven to be positive in the past, increasing performance, promoting the growth of beneficial microbial populations and enhancing intestinal health^20–25,28,29^. In this study, XOS was provided to suckling piglets at very low doses (30 to 60 mg/day) to explore their likely effects as stimbiotics, with the aim of modulating the early-colonization of the gut microbiota and the establishment of a higher fibrolytic environment with age. Inclusion of soluble fiber, such as oligosaccharides, in the diet of newly weaned piglets has been considered a risk factor for pig health and growth in some reports, especially under poor sanitary conditions, due to a limited digestive capacity of piglets^30^. In this trial, XOS did not influence piglet’s performance during suckling (d28) nor the post-weaning period (d40), indicating that their digestive system tolerated the supplementation of low doses of XOS during this period without conditioning their performance. No differences were also observed on the SCFAs concentration in feces.

### Evolution of gut microbiota from suckling to nursery phase

The process of weaning is associated with a combination of environmental, social and dietary stressors that are known to generate changes in the piglet’s gut microbial ecosystem^1,2^. The high throughput sequencing of the 16S RNA gene from the feces of the piglets revealed changes in the microbial diversity and composition between the end of lactation and the nursery period. The number of observed OTUs, that is an indicator of richness, and the Shannon index, that reflects richness and evenness, increased in the post-weaning period. This result agrees with other studies^31,32^ that reported an increase in alpha diversity indexes after weaning transition, suggesting the maturation of the gut microbiota during that period. Alpha diversity is considered an indicator of gut ecosystem maturation, because a higher diversity of bacterial species suggests a higher “functional redundancy” that helps the microbial ecosystem maintain its resilience, resistance and stability after environmental stresses^33,34^. Beta diversity analysis corroborated that pre-weaning and post-weaning gut populations had different microbiota structures.

The microbial composition of piglets’ feces from this study are in the line with pig microbiota described in the literature^35^. Firmicutes and Bacteroidetes were the dominant phyla in piglets feces regardless of age, in agreement with other studies^32,35–39^. Firmicutes was the main phylum in the whole period of the study and increased its relative abundance in the nursery period. The abundance of Proteobacteria drastically decreased in the nursery period, as has been reported previously^31,36,38^. Proteobacteria include multiple opportunistic pathogens, thus, its reduction might reflect the maturation of gut microbiota during the weaning transition. From lactation to the nursery period, at the family level, the relative abundance of *Bacteriodaceae* abruptly decreased at the same time that *Ruminococcaceae*, *Lachnospiraceae* and *Prevotellaceae* increased. These shifts might be explained by the diet change from sow’s milk during lactation to a solid plant-based diet as reported in other studies^31,32,36,39,40^. *Bacteroideaceae* members have the ability to metabolize complex oligosaccharides present in the sow milk^41^, while *Ruminococcaceae*, *Lachnospiraceae* and *Prevotellaceae* families are adapted to break down oligosaccharides and polysaccharides present in the nursery feed^42^.

Differences observed in the fecal microbial communities between pre- and post-weaning were concurrent with relevant changes in the predicted functionality of the microbiota obtained from PICRUSt2 analysis. This indicates that the different gut populations found in each period also had a distinct metabolic profile. Indeed, the expansion of fibrolytic bacteria in the nursery period was correlated with an increase in total SCFAs, VFAs, acetic acid, propionic acid, butyric acid and lactic acid concentrations in feces observed in the same period, as has been described in other studies^43^.

### Effects of XOS supplementation on gut microbiota

The main objective of the present study was to explore the effect of providing low doses (30 to 60 mg/day) of XOS to suckling piglets in their fecal microbiota at the end of lactation and in the nursery period. Provision of XOS to pigs has been extensively shown to modulate their gut microbiota, promoting the growth of *Bifidobacterium* and other beneficial fiber-degrading and SCFA-producing bacteria^20,22,29^, even at very low doses^21,26^. In this study animals supplemented with XOS showed a higher OTU richness in the fecal microbiota, suggesting that XOS promoted an earlier microbial maturation. In addition, beta diversity analysis revealed that microbial populations of the XOS group tended to differ from the CON group in the nursery period.

The effect of XOS in the hindgut microbiota of the piglets at the end of lactation was characterized by an increase in the relative abundance of some taxa associated to fiber fermentation such as *Eubacteriaceae* at the family level and *Ruminococcaceae* UCG-013, *Prevotella* 7, *Ruminiclostridium* 5, *Ruminoccocus* 1 and *Ruminiclostrium* 9 at the genus level. These groups are considered beneficial for the host health due to their capacity to produce SCFAs through the fermentation of complex polysaccharides, and in addition, some of them have been associated to lower diarrhea incidence in suckling pigs^44^. Furthermore, members of *Eubacteriaceae* family and *Prevotella* genus had demonstrated positive correlations with elevated pig growth performance^45–47^. Supplementation of XOS also increased the abundance of *Erysipelotrichaceae*. Members of the *Ruminococcaceae*, *Prevotellaceae* and *Erysipelotrichaceae* families are considered typical post-weaning bacteria^31,32^, and their enrichment in XOS-supplemented piglets at the end of the lactation phase suggests that XOS supplementation accelerates gut microbiota maturation. In contrast, *Blautia* relative abundance was lower in XOS-supplemented pigs even though it is considered as a genus with probiotic characteristics, capable of fermenting carbohydrates for SCFAs production^48^.

At 12 days post-weaning, differences in the relative abundances of specific taxa were also observed. Like at the end of lactation, XOS supplementation increased the abundances of genera related to fiber fermentation and SCFAs production in the nursery period, such as *Bacteroides*, *Moryella*, *Ruminiclostridium* 6 and *Lachnospiraceae* NK4A136. Interestingly *Bacteroides* was found to be 6.75 log-twofold higher in XOS-supplemented piglets compared to CON. *Bacteroides* are major primary degraders of oligo- and polysaccharides with a close relationship with XOS, containing the most expanded glycolytic gene repertoires that target xylan degradation^49^. In fact, different in vitro tests have demonstrated that XOS stimulates *Bacteroides* growth in the microbial ecosystem^16,50^. The abundance of *Lachnospiraceae* in mice fed a high-fat diet was increased by XOS, increasing the formation of butyric acid in the cecum^17^.

Functional profile predictions demonstrated differences in the pathway abundance between CON and XOS in both periods. However, more differences were found during the nursery period, which is consistent with the tendency for the microbial structure of both treatments to be different in the nursery period. The higher relative abundance of the predicted pathway “pyruvate fermentation to butanoate” found in XOS-supplemented pigs in the nursery period is in agreement with the higher presence of fermenters producing SCFAs, including butyrate, such as *Bacteroides*, *Moryella*, *Ruminiclostridium* 6 and *Lachnospiraceae* NK4A136. Actually, SCFAs decrease the pH in the intestine, promote gastrointestinal motility and inhibit the proliferation of opportunistic pathogens^51^. Among them, butyrate is the preferred energy source used by colonocytes, which is involved with the maintenance of the gut barrier function and has been related in anti-inflammatory pathways^19^. Nevertheless, neither the increase of certain SCFA-producing bacterial populations observed in the XOS-supplemented piglets nor the increase in the predicted pathway of butyrate production in the nursery period resulted in any change in fecal SCFAs concentrations. It is more likely to observe changes in the production of SCFA using in vitro tests including XOS^16,52^, while there is more uncertainty to see such effects from in vivo studies^20,28,29^. Most SCFAs (95%) produced by the microbiota are quickly absorbed by the mucosa, while only 5% are excreted in the feces^51^. In fact, several studies have reported that increases in SCFAs concentration in portal blood could not be predicted from the concentrations in the intestinal lumen^53,54^. Therefore, SCFAs concentration found in the feces might have a limited value in identifying their production in the intestine and future studies should use cecum or intestinal digesta for its determination instead.

The nursery diet offered to both treatments did not include XOS but it contained xylanases, which are expected to hydrolyze dietary arabinoxylan molecules and release XOS in the gut of post-weaned piglets. In spite of this, the animals supplemented with XOS during the suckling period still maintained differences in microbial populations, structure and functions in the nursery period compared to CON animals, which confirms the hypothesis that an early-supplementation of suckling piglets with XOS can accelerate the establishment of a different microbial population with age. The results of this study agree with Bai et al.^28^, who studied the effect of adding XOS in the creep feed of suckling piglets, and showed an increase in the predicted microbial ability for carbohydrate digestion and absorption capacity in the feces at weaning and a higher xylanase activity at 27 days after weaning, even though they were fed the same diet after weaning. Several studies evaluating dietary interventions, such as the supplementation of prebiotics, fiber or functional ingredients to modulate the functioning of the gastrointestinal tract in suckling piglets have been published^11^, but only a few of them have investigated the long-term effects post-weaning. These studies described a disappearance of the differences in the gut microbial populations generated during the suckling period after weaning occurs^12,55^. The process of gut microbiota maturation supposes a homogenization and stabilization of its structure and populations that might blur the effects of certain interventions made during suckling^31,32^. The persistence of the effects of XOS in the gut microbiota after weaning emphasizes its potential in modulating the early microbial colonization of piglets’ gut and suggests it might have effects on the long-term performance and health.

In summary, the supplementation of low doses (30 to 60 mg/day) of XOS to piglets during the suckling period promoted the growth of fiber-degrading and SCFA-producing microbial populations in the hindgut. The effects of supplementing XOS only during lactation are still observed in the nursery period, when all piglets were on the same diet and environment, suggesting that XOS conditions the establishment of a differential microbiota population in the long term.

## Methodology

The experimental procedures used were approved by the Ethical Committee on Animal Experimentation of the Universitat Autònoma de Barcelona (CEAAH 3817), and are in full compliance with national legislation following the EU-Directive 2010/63/EU for the protection of animals used for scientific purposes and designed in compliance with the ARRIVE guidelines.

### Animals, housing and diet

The study was conducted in the farrowing room of a commercial farm from day 7 of lactation to the day of weaning (d28) and in the nursery unit of the same farm from weaning to 12 days post-weaning (d40). Ninety commercial male and female piglets ((Landrace × Large White) × Duroc) from 15 litters were included in the trial. On d7 of lactation, six piglets from each litter with an average body weight (BW; 2.91 ± 0.42 kg) were selected for the experiment, ear-tagged and divided into two experimental treatments (3 piglets/treatment/litter) according to its BW on d7 and sex. From d7 to d27 of lactation, piglets in the control treatment (CON, *n* = 45) were individually administered 5 mL of water once a day while piglets in the supplemented group (XOS, *n* = 45) individually received 5 mL of a solution containing 30 mg XOS (AB Vista, Marlborough, UK) obtained from corn cob with a purity of 35% of XOS from d7 to d14 and 60 mg XOS from d15 to d27 by oral gavage. Sows and their litters were housed in individual farrowing pens (2.6 × 1.8 m^2^), in a partially slatted floor with a heated floor pad for piglets, equipped with a farrowing crate, an individual feeder and nipple drinkers for sows and piglets. The temperature in the farrowing room was automatically controlled. Water and feed were offered ad libitum to the sows while piglets were offered only water. Piglets did not have access to creep feed during the lactation period.

At weaning (d28), supplementation ceased and piglets were moved to the nursery unit without transport. Piglets were randomly allocated in 6 pens blocked by sex (3 female pens and 3 male pens; 15 animals/pen), mixing animals from CON and XOS in the same pens. Each pen (3.20 m^2^) was equipped with two commercial pan feeders (Maxi hopper, Rotecna, Spain) and a nipple bowl drinker to provide ad libitum access to feed and water. The floor was completely slatted, and the temperature and ventilation rates were controlled using central and forced ventilation with an automatic cooling system. All animals were fed a common conventional pre-starter diet formulated to contain 2470 kcal NE/kg, 18.8 CP/kg and 1.425% digestible Lys (Table 4) and to meet the requirements for maintenance and growth of newly weaned piglets^56^. No XOS were supplemented in the pre-starter diet.

**Table 4:** Nursery diet offered to the animals included in the trial.

Item	Nursery diet
**Ingredient, %**	
Wheat	23.4
Extruded barley	20.0
Acid whey	10.0
Corn	10.0
Soybean protein concentrate	8.3
Soybean meal heat processed	7.0
Dextrose	4.0
Fish meal	3.0
Spray-dried plasma	3.0
Milk whey 50% fat	2.5
Nucleus^a^	2.0
Beet pulp	2.0
Lard	1.85
Mono calcium phosphate	0.72
l-Lysine sulphate	0.72
Vitamin-mineral premix^b^	0.4
l-Threonine	0.31
Calcium carbonate	0.31
dl-Methionine	0.29
Salt	0.20
l-Valine	0.08
**Calculated composition**	
NE, kcal/kg	2470
Ash, %	3.1
Crude protein, %	18.8
Ether extract, %	6.8
Crude fiber	3.1
Starch	30.1
Calcium, %	0.542
Total P, %	0.620
Digestible P, %	0.496
**Digestible amino acids**	
Lys, %	1.425
Met, %	0.562
Met + Cys, %	0.894
Thr, %	1.027
Trp, %	0.257

^a^Basic composition of the nucleus: yogurt, extruded soybean, micronized carob meal, nucleotides, hyperimmune egg and endo-1,4 beta-xylanase (420 UI/kg).

^b^Provided per kilogram of diet: 12,000 IU of vitamin A (acetate); 2000 IU of vitamin D3 (cholecalciferol); 250 IU of vitamin D (25-hydroxicholecalciferol); 75 mg of vitamin E; 2 mg of vitamin K3; 3 mg of vitamin B1; 7 mg of vitamin B2; 7.33 mg of vitamin B6; 15 mg of vitamin B12; 17 mg of d-pantothenic acid; 45 mg of niacin; 0.2 mg of biotin; 1.5 mg of folacin; 80 mg of Fe (chelate of amino acids); 100 mg of Zn (chloride); 12.5 mg Zn (chelate of amino acids); 12.5 mg of Mn (chloride); 0.3 mg of Se (inorganic); and 2.04 mg of BHT.

### Performance measurements and sample collection

Piglets were individually weighed on d7, d28 (weaning) and d40 of age. The ADG was calculated for the experimental period. At weaning, fecal samples were collected from a piglet with a medium body weight from each litter and treatment (*n* = 15). Same piglets were used to obtain fecal samples on d40. In both periods, an aliquot of the feces was stored in 2 mL sterile cryotubes, snap frozen in dry ice, and afterward kept at – 80 °C for analyses of fecal microbiota. Another aliquot was stored in the Biofreezer tubes (Alimetric Diagnostics, Espoo, Finland) following the recommended protocol by the manufacturer for the analysis of SCFA. Considering that the present study had an exploratory will, both microbiota and SCFA characterization were performed in fecal samples and not in intestinal digesta to avoid euthanizing animals, following the principle of the three R’s (Replacement, Reduction and Refinement) for more ethical use of animals in scientific research.

### Short-chain fatty acid analysis

The fecal SCFA were analysed as free acids by gas chromatography. Briefly, 1 mL of H_2_O was added to 1 g of ceca content, and 1 mL of a solution containing 20 mmol/L pivalic acid was incorporated as an internal standard. Afterwards, 1 mL of perchloric acid was added, and SCFA were extracted by shaking the mixture for 5 min. After centrifugation, 50 μL of 4 mol KOH in 500 μL of supernatant were added to precipitate the perchloric acid in the supernatant. Saturated oxalic acid was added after 5 min, the mixture was incubated at 4 °C for 60 min and then centrifuged at 18,000×*g* for 10 min. The chromatography procedure used a glass column packed with 80/120 Carbopack B-DA/4% Carbowax 20 mol stationary phase (Supelco, Bellefonte, PA), using a flame ionization detector and helium as the carrier gas^57^. The acids measured were lactic acid and VFA which in turn comprised of acetic, propionic, butyric, iso-butyric, 2-methyl-butyric and iso-valeric acids. The sum of isobutyrate, 2-methyl butyrate and isovalerate results in branched-chain fatty acids (BCFA).

### 16S rRNA gene sequencing

The fecal samples stored at – 80 °C were used for the determination of the composition and structure of microbial communities present through a 16S ribosomal RNA gene sequence-based analysis. Bacterial DNA was extracted from 250 mg of each fecal sample using the commercial MagMAX CORE Nucleic Acid Purification Kit 500RXN (Thermo Fisher, Barcelona, Spain) following the manufacturer's instructions. In order to ensure the quality of the analysis, a negative control and a Mock Community control (Zymobiomics Microbial Community DNA) were included. Samples were amplified using specific primers for the V3-V4 regions of the 16S rRNA DNA (F5′-TCGTCGGCAGCGTCAGATGTGTATAAGAGACAGCCTACGGGNGGCWGCAG-3′, R5′GTCTCGTGGGCTCGGAGATGTGTATAAGAGACAGGACTACHVGGGTATCTAATCC-3′)^58^. The library preparation was performed in Microomics Systems SL (Barcelona, Spain). The Illumina Miseq sequencing 300 × 2 approach was used and amplification was performed after 25 PCR cycles.

For sequencing data bioinformatics, the sequence reads generated were processed using QIIME version 2019.4 software^59^. The software package DADA2 was used for primer trimming, quality filtering, denoising, pair-end merging, and amplicon sequence variant calling (ASV, i.e., phylotypes) using qiime dada2 denoise-paired method^60^. Also, Q20 was used as a quality threshold to define read sizes for trimming before merging (parameters: -p-trunc-len-f and -p-trunc- len-r). Reads were truncated at the place when the 25th percentile Phred score felt below Q20 for both forward and reverse reads. After quality filtering steps, the average sample size of reads was resolved and phylotypes were detected. To even sample sizes for the diversity analysis using qiime diversity core metrics-phylogenetic pipeline, ASV tables were subsampled without replacement. Bray–Curtis distances were calculated to compare community structure. Taxonomic assignment of phylotypes was performed using a Bayesian Classifier trained with Silva V4 database version 132 (99% OTUs full-length sequences)^61^.

### Functional predictions

The functional potential of the gut microbiota was explored by inferring metagenomics functionality from the 16S rRNA gene sequencing data using the Phylogenetic Investigation of Communities by Reconstruction of Unobserved States (PICRUSt2) software^62^. Individual gene-family copy numbers for each ASVs was estimated after placing sequences into a reference phylogeny tree containing more than 20,000 full 16SrRNA genes from bacterial and archaeal genomes from the Integrated Microbial Genomes (IMG) database^63^. Afterwards, ASVs are corrected by their 16S rRNA gene copy number, and pathway abundances are inferred on the basis of structured pathway against the Kyoto Encyclopedia of Genes and Genomes (KEGG)^64^ orthologs (KOs) and Enzyme Commission numbers (EC numbers) databases.

### Statistical analysis

Growth performance data and SCFAs concentration data were analyzed with ANOVA using the general linear model (GLM) procedure of statistical package SAS (version 9.4, SAS Institute Inc., Cary, NC). Growth performance was analyzed as a complete randomized design and SCFAs concentration as a factorial arrangement (treatment × day). Normality and homoscedasticity were checked with Shapiro–Wilk test using the univariate procedure and Levene’s test using the generalized linear model procedure, respectively. The LSMeans statement was used to calculate mean values for each parameter.

The statistical analysis of the fecal microbiota was performed in open-source software R v4.0.3 (R Foundation for Statistical Computing, Vienna, Austria). Alpha diversity was calculated using vegan package^65^ from raw counts (OTU level) including observed OTUs and Shannon index. An ANOVA test was performed to test group differences for alpha diversity. Principal coordinates analysis (PCoA) were calculated using beta diversity distance matrices (Bray–Curtis). Permutational multivariate analysis of variance (PERMANOVA) was used to test the effects of day and treatment, and their interaction on the Bray–Curtis distance between samples. Microbial diversity was analyzed as a factorial arrangement taking treatment and sampling day as main factors and main effects are discussed for responses in which interaction was not significant. Differential abundance analysis was performed using the metagenomeseq package^66^ to examine differences in phylum level, family level, genus level and predicted pathway data. Separate analysis were performed for each sampling day to compare CON and XOS treatments and another one to compare between the two sampling days, independently of the treatment. A cumulative sum scaling (CSS)^67^ normalization of the raw counts was performed and a zero-inflated Gaussian mixture model was used for the analysis. Relative abundances were used to plot taxon abundances at phylum and family level for each sampling day. Log_2_-fold changes were calculated using relative abundances for the taxonomical groups that showed different abundance between treatments at each sampling day. *P*-values were corrected by the false-discovery rate. The pig was considered the statistical unit in all analyses and statistical significance and tendencies were considered at *P* ≤ 0.05 and 0.05 < *P* ≤ 0.10, respectively.

## Supplementary Information

Supplementary Table S1.

Supplementary Table S2.

Supplementary Table S3.

Supplementary Table S4.

## Data Availability

The raw sequencing data employed in this article has been submitted to the NCBI’s sequence read archive (https://www.ncbi.nlm.nih.gov/sra); BioProject: PRJNA824854.
